# Enhancement of radiosensitivity in human glioblastoma cells by the DNA N-mustard alkylating agent BO-1051 through augmented and sustained DNA damage response

**DOI:** 10.1186/1748-717X-6-7

**Published:** 2011-01-19

**Authors:** Pei-Ming Chu, Shih-Hwa Chiou, Tsann-Long Su, Yi-Jang Lee, Li-Hsin Chen, Yi-Wei Chen, Sang-Hue Yen, Ming-Teh Chen, Ming-Hsiung Chen, Yang-Hsin Shih, Pang-Hsien Tu, Hsin-I Ma

**Affiliations:** 1 Graduate Institutes of Life Sciences, National Defense Medical Center & Department of Neurological Surgery, Tri-Service General Hospital, Taipei, Taiwan; 2 Department of Medical Research and Education, Taipei Veterans General Hospital, Taipei, Taiwan; 3 Institute of Pharmacology, National Yang-Ming University, Taipei, Taiwan; 4 Institute of Clinical Medicine, School of Medicine, National Yang-Ming University, Taipei, Taiwan; 5 Institute of Biomedical Sciences, Academia Sinica, Taipei, Taiwan; 6 Department of Biomedical Image and Radiological Sciences, School of Biomedical Science and Engineering, National Yang-Ming University, Taipei, Taiwan; 7 Cancer Center, Taipei Veterans General Hospital, Taipei, Taiwan; 8 Department of Neurosurgery, Neurological Institute, Taipei Veterans General Hospital, Taipei, Taiwan

## Abstract

**Background:**

1-{4-[Bis(2-chloroethyl)amino]phenyl}-3-[2-methyl-5-(4-methylacridin-9-ylamino)phenyl]urea (BO-1051) is an N-mustard DNA alkylating agent reported to exhibit antitumor activity. Here we further investigate the effects of this compound on radiation responses of human gliomas, which are notorious for the high resistance to radiotherapy.

**Methods:**

The clonogenic assay was used to determine the IC_50 _and radiosensitivity of human glioma cell lines (U87MG, U251MG and GBM-3) following BO-1051. DNA histogram and propidium iodide-Annexin V staining were used to determine the cell cycle distribution and the apoptosis, respectively. DNA damage and repair state were determined by γ-H2AX foci, and mitotic catastrophe was measure using nuclear fragmentation. Xenograft tumors were measured with a caliper, and the survival rate was determined using Kaplan-Meier method.

**Results:**

BO-1051 inhibited growth of human gliomas in a dose- and time-dependent manner. Using the dosage at IC_50_, BO-1051 significantly enhanced radiosensitivity to different extents [The sensitizer enhancement ratio was between 1.24 and 1.50 at 10% of survival fraction]. The radiosensitive G_2_/M population was raised by BO-1051, whereas apoptosis and mitotic catastrophe were not affected. γ-H2AX foci was greatly increased and sustained by combined BO-1051 and γ-rays, suggested that DNA damage or repair capacity was impaired during treatment. *In vivo *studies further demonstrated that BO-1051 enhanced the radiotherapeutic effects on GBM-3-beared xenograft tumors, by which the sensitizer enhancement ratio was 1.97. The survival rate of treated mice was also increased accordingly.

**Conclusions:**

These results indicate that BO-1051 can effectively enhance glioma cell radiosensitivity *in vitro *and *in vivo*. It suggests that BO-1051 is a potent radiosensitizer for treating human glioma cells.

## Background

Malignant gliomas account for approximately 30% of all intracranial tumors, and of them, glioblastoma multiforme (GBM) is considered as the most frequent and aggressive type. Removal of GBM by surgical resection is usually not feasible due to the highly diffuse infiltrative growth and recurrence rate [1]. A multicenter study has shown that addition of concurrent temozolomide (TMZ) to radical radiation therapy improves the survival in patients who suffered from GBM [2,3]. These studies have demonstrated an improvement for patients who received TMZ, compared to those who did not, in the median survival time and in the 2-year survival rate (14.6 vs. 12 months, 27% vs. 10%, respectively). Unfortunately, the survival rate remains low using TMZ, and it prompts investigators to seek new and more effective chemotherapeutic agents for the treatment of malignant gliomas.

DNA alkylating agents are used widely for treatment of a variety of pediatric and adult cancers because the cytotoxic effects of these agents can directly modify DNA and cause DNA lesions [4]. However, the development of new alkylating N-mustard agents is slow due to their low tumor specificity, high chemical reactivity and an induction of bone marrow toxicity [5,6]. To overcome these drawbacks, one strategy has been to design DNA-directed alkylating agents by linking the alkylating pharmacophore to the DNA-affinity molecules (e.g., DNA intercalating agents, DNA minor groove binder) [7,8]. In most cases, the DNA-directed alkylating agents have more selective, cytotoxic and potential than the corresponding untargeted derivatives [8-10]. Among these agents, the compound BO-0742 exhibited significant cytotoxicity (107-fold higher) on human lymphoblastic leukemic cells than its parent analogue 3-(9-acridinylamino)-5-hydroxymethylaniline [9,11].

BO-0742 was found to have a potent therapeutic efficacy against human leukemia and solid tumor cell growth *in vitro*. Also, it has a good therapeutic index with leukemia being 10-40 times more sensitive than hematopoietic progenitors. Administration of BO-0742 at an optimal dose schedule, based on its pharmacokinetics, significantly suppressed the growth of xenograft tumors in mice bearing human breast and ovarian cancers. However, BO-0742's bioavailability is low because it has a narrow therapeutic window and is chemically unstable in mice (half-life < 25 min) [12]. To improve the poor pharmacokinetics of BO-0742, we have recently synthesized a series of phenyl N-mustard-9-anilinoacridine conjugates via a urea linker [13,14]. Of these agents, BO-1051 was found to be more chemically stable than BO-0742 in rat plasma (54.2 vs. 0.4 h). BO-1051, an agent capable of inducing marked dose-dependent levels of DNA interstrand cross-linking (ICLs), revealed a broad spectrum of anti-cancer activities *in vitro *without cross-resistance to taxol or vinblastine. Due to BO-1051's hydrophobic ability, it can penetrate through the blood-brain barrier to brain cortex. BO-1051 has been shown to possess therapeutic efficacy in nude mice bearing human breast MX-1 tumors and human glioma *in vivo *[14]. Interestingly, we found that obvious tumor suppression was observed in mice and sustained over 70 days without relapse [14]. The results indicated that BO-1051 was more potent than cyclophosphamide with low toxicity to the host (15% body-weight drop) suggesting that this agent is a promising candidate for preclinical studies.

Given that radiotherapy is considered to be the most effective adjuvant treatment with surgery, we tested if the therapeutic ability of BO-1051 could be translated into antitumor activity. In this study, we investigated the effects of BO-1051 on the radiosensitivity of a panel of three human glioma cell lines, and we found that treatment with BO-1051 at nanomolar concentrations sensitizes the glioma cells to radiation-induced cellular lethality. These data indicate that BO-1051 enhances tumor radiosensitivity *in vitro *and *in vivo*. Moreover, this sensitization correlates with its enhancement arrest in the radiosensitive cell cycle phase and the delayed dispersion of phosphorylated histone H2AX (γ-H2AX) foci, which suggests an inhibition of the repair to the DNA double-strand breaks (DBSs).

## Materials and Methods

### Cell lines and treatment

**Figure 1:**
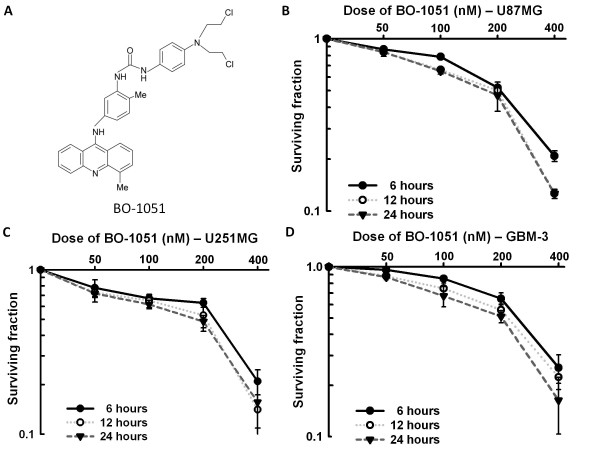
**Clonogenic survival of human glioma cells treated with BO-1051**. (A) Chemical structure of 1-{4-[bis(2-chloroethyl)amino]phenyl}-3-[2-methyl-5-(4- methylacridin-9-ylamino)phenyl]urea (BO-1051). (B) U87MG, (C) U251MG and (D) GBM-3 cells were exposed to escalating doses (50-400 nM) of BO-1051 or vehicle (DMSO). At 6, 12 and 24 h after the addition of BO-1051, the BO-1051- containing medium was removed, rinsed, and then fed with fresh growth media. Colony- forming efficiency was determined 10-14 days later, and the survival fractions of BO-1051-treated cells were calculated after normalizing for the plating efficiencies of untreated cells. Points: mean for at least 3 independent experiments; bars, SD.

This research followed the tenets of the Declaration of Helsinki. All samples were obtained after patients provided informed consent. The study was approved by the Institutional Ethics Committee/Institutional Review Board of Tri-Service General Hospital. The commercial available U87MG, and U251MG glioma cell lines as well as primary GBM cell line (GBM-3), which was isolated from tumor sample obtained from patient undergoing surgery for a GBM (World Health Organizing Grade 4 astrocytoma), were grown as attached monolayers in 75-cm^2 ^flasks in DMEM media (Invitrogen) supplemented with glutamate (5 mmol/L) and 10% fetal bovine serum. Cells were incubated at the exponential growth phase in humidified 5% CO_2_/95% air atmosphere at 37℃. The GBM-3 cells used for the experiments had already undergone > 100 passages. 1-{4-[bis(2-chloroethyl)amino]phenyl}-3-[2-methyl-5- (4-methylacridin-9-ylamino)phenyl]urea (BO-0151, Figure 1A) was dissolved in DMSO to a stock concentration of 5 mM and stored at -20℃. Gamma radiation (ionizing irradiation) was delivered with a T-1000 Theratronic cobalt unit (Theratronic International, Inc., Ottawa, Canada) at a dose rate of 1.1 Gy/min (SSD = 57.5 cm).

### Assay of BO-1051 cytotoxicity

For these studies, a specified number of single cells were seeded into a 25-T flask, and after 6 h, to allow for cell attachment (but no division), the cells were treated with 0, 50, 100, 200 or 400 nM BO-1051. At 0, 6, 12 and 24 h after the BO-1051 addition, the BO-1051-containing medium was removed; the cells were washed with sterile PBS, and fresh media was added. After 10 to 14 days of incubation, colonies were fixed with methanol and stained with Giemsa. The number of colonies containing at least 50 cells was determined, and the plating efficiency (PE) and surviving fractions (SF) were calculated. The SF of cells exposed to × nM BO-1051 for t h was calculated as [15]:

$${\text{SF}}_{xnM,thr}=\frac{{\text{PE}}_{\text{xnM,thr}}}{{\text{PE}}_{\text{0nM,thr}}}$$

This protocol was used in an attempt to eliminate any effects of trypsinization on post-treatment or post-irradiation signaling/recovery processes [16-20]. Moreover, this protocol allows for the irradiation of single cells but not microcolonies, which eliminates the confounding parameter of multiplicity and its effects on the radiosensitivity.

### Combination of BO-1051 and irradiation

After allowing the cells time to attach, the culture medium was then replaced with fresh medium that contained 200 nM BO-1051, and the flasks were irradiated 24 h later. Immediately after irradiation, the growth media was aspirated, and fresh media was added. Colonies were stained with Giemsa 10 to 14 days after seeding. Survival curves were then generated after normalizing for the amount of BO-1051-induced cell death. The radiation SF of cells pretreated with × nM BO-1051 was calculated as [15]:

$${\text{SF}}_{\text{xnM,DGy}}=\frac{{\text{PE}}_{\text{xnM,DGy}}}{{\text{PE}}_{\text{xnM,0Gy}}}$$

The combined therapeutic effects based on drug and ionizing irradiation was obtained by the survival fractions measured by separate treatment as reported previously [21]. The expected effect by two separate treatments was determined by the formula SF_(Drug) _× SF_(Rad)_, which was compared to the observed survival fraction.

### Cell-cycle analysis

After treatment, cells were prepared for fluorescence-activated cell sorting (FACS) to assess the relative distribution in the respective phases of the cell cycle. Cells were harvested 24 h after of treatment with BO-1051, pelleted by centrifugation, re-suspended in PBS, fixed in 70% ethanol and stored at -20℃. Immediately before flow cytometry, the cells were washed in cold PBS (4℃), incubated in Ribonuclease A (Sigma) for 20 min at room temperature, labeled by adding an equal volume of propidium iodide solution (100 μg/ml) and incubated in the dark for 20 min at 4℃. These samples were measured (20,000 events collected from each) in a FACSCalibur cytometer (BD FACS Caliber; Mountain View, CA). The data shown are for one experiment, but the results were reproduced and confirmed in at least three identical experiments.

### Annexin V-PI apoptosis assay

To evaluate apoptosis as a mechanism of cell death, approximately 2 × 10^6 ^cells were plated in 100-mm petri dishes. Cells were exposed to 200 nM or higher concentration (1.2 μM) of BO-1051 prior to irradiation and were stained at 24 and 48 h postirradiation (2 Gy). Both adherent and detached cells were collected, centrifuged, and double stained with Annexin V-FITC and propidium iodide (PI). Apoptotic cells were quantified with flow cytometry using a FACSCalibur cytometer (BD FACS Caliber, Mountain View, CA).

### Immunofluorescent staining for γ-H2AX

Cells were treated with or without BO-1051 for 24 h prior to irradiation (2 Gy) and fed with BO-1051-free medium, and the average number of foci per cell was measured beginning at 1 h after irradiation and followed thereafter for 24 h. At specified times, the media were aspirated and cells were fixed in 1% paraformaldehyde for 10 min at room temperature. Paraformaldehyde was aspirated, and the cells were treated with a 0.2% NP40/PBS solution for 15 min. Cells were then washed in PBS twice, and the anti-γH2AX antibody was added at a dilution of 1:500 in 1% BSA and incubated overnight at 4℃. Again, the cells were washed twice in PBS before incubating in the dark for 1 h with a FITC-labeled secondary antibody at a dilution of 1:100 in 1% BSA. The secondary antibody solution was then aspirated, and the cells were washed twice in PBS. The cells were then incubated in the dark with PI (1 μg/ml) in PBS for 30 min, washed twice, and coverslips were mounted with an anti-fade solution (Dako Corp.; Carpinteria, CA). Slides were examined with a confocal fluorescent microscope (Wetzlar, Germany). Images were captured by a Photometrics Sensys CCD camera (Roper Scientific; Tucson, AZ) and imported into the IP Labs image analysis software package (Scanalytics, Inc.; Fairfax, VA) running on a Macintosh G3 computer. For each treatment condition, γ-H2AX foci were determined in at least 150 cells.

### *In vivo *tumor model

Six-week-old female nude mice were used in these studies. Mice were caged in groups of five or less, and all animals were fed a diet of animal chow and water ad libitum. All procedures involving animals were performed in accordance with the institutional animal welfare guidelines of the Taipei Veterans General Hospital. Tumors were generated by injecting 5 × 10^6 ^GBM-3 cells subcutaneous (s.c.) into the right hind leg. Irradiation was performed using a T-1000 Theratronic cobalt unit (Theratronic International, Inc.; Ottawa, Canada) irradiator with animals restrained in a custom jig.

### Tumor growth delay assay

The tumor re-growth delay assay measures the time required for a tumor to reach a given size post-treatment. When tumors grew to a mean volume of ~150 mm^3^, mice were randomly assigned to one of four treatment groups: vehicle control (14 animals), BO-1051 (12 animals), 4 Gy irradiation (9 animals), or combined BO-1051 and radiation (8 animals). BO-1051 treatment was performed, which consisted of an intraperitoneal (i.p.) injection protocol of 50 mg/kg administered at 3-day intervals over a 6-day period (3 injections on days 0, 3, 6; Q3D × 3). For irradiation, unanesthetized animals were immobilized in a lead jig that allowed for the localized irradiation of the implanted tumors. Gamma radiation was delivered by a T-1000 Theratronic cobalt unit (Theratronic International, Inc.; Ottawa, Canada) at a dose rate of 1.1 Gy/min (SSD = 57.5 cm). For the BO-1051-plus-radiation group, BO-1051 (50 mg/kg) was delivered via i.p. injection on days 0, 3, and 6, with day 0 being the day of randomization. Radiation (4 Gy) was delivered to animals restrained in a custom lead jig 24 h after the first injection of BO-1051 (day 1 after randomization). Tumor volume is a critical parameter in determining radiation-induced growth delay with smaller tumors appearing more radiosensitive. To ensure BO-1051-induced growth delay did not bias the results of the combination treatment (BO-1051 plus 4 Gy), it was important that the two irradiated groups (4 Gy and BO-1051 plus 4 Gy) received radiation when the tumors were approximately the same size. To obtain tumor growth curves, perpendicular diameter measurements of each tumor were made every day with digital calipers, and the volumes were calculated using the formula for volume of an ellipsoid: 4Π/3 × *L*/2 × *W*/2 × *H*/2, where *L *= length, *W *= width, and *H *= height. The time for the tumor to grow again to ten times the initial volume (about 1500 mm^3^) was calculated for each animal. Absolute tumor growth delay was calculated as the number of days for the treated tumors to reach ten times the initial tumor volume minus the number of days for the control group to reach the same size.

The mean size of tumors receiving the combination treatment was compared to the mean size of tumors in mice from each of the other groups (receiving vehicle control, radiation alone, or BO-1051 alone). The analysis was done on day 42 after the treatment started because this was the last day that all animals were still alive. Time to treatment failure (TTF) was defined as the time from the initiation of treatment (experimental or control) to the time a tumor was severely necrotic or had reached a volume > 1500 mm^3^. Normalized tumor growth delay is defined as the time in days for tumors to reach 10 times the initial volume in mice treated with the combination of BO-1051 and radiation minus the time in days for the tumors to reach 10 times the initial volume in mice treated with BO-1051 only, which was 6.7 days (i.e., 16 minus 9.3 days).

### Statistical analysis

The results are reported as mean ± SD. Statistical analysis was performed using a Student's t-test, one-way ANOVA test or two-way ANOVA test followed by Tukey's test, as appropriate. A *P *< 0.05 was considered to be statistically significant.

## Results

### Determination of the cytotoxicity of BO-1051 on different human glioma cell lines

To determine the effects of BO-1051 on glioma cell cytotoxicity by clonogenic survival, MTT assay was performed in a panel of 3 human malignant glioma cell lines (U87MG, U251MG and GBM-3). The IC_50 _(concentration resulting in cell viability of 50% of control) values of BO-1051 for U87MG, U251MG and GBM-3 cells were 2.7, 2.5 and 1.5 μM, respectively. However, the clonogenic survival analysis showed little or no colony formation for 24 h post-exposure to the concentrations of BO-1051 > 400 nM. We found that the appropriate dosage range of BO-1051 for colony formation in these glioma cell lines was between 50 and 400 nM. The cytotoxicity of U87MG, U251MG and GBM-3 cells were significantly influenced by BO-1051 in a time-dependent and dose-dependent manner. The 24-h treatment of 200 nM BO-1051 resulted in SFs of 0.470 ± 0.091, 0.485 ± 0.041 and 0.510 ± 0.042 for U87MG, U251MG, and GBM-3, respectively (Figure 1). Because approximately 50% of survival fractions were reached using 200 nM BO-1051 treatments on each glioma cells at 24 h, we chose this dose for the following experiments.

### Enhancement of radiosensitivity in glioma cells by BO-1051

**Figure 2:**
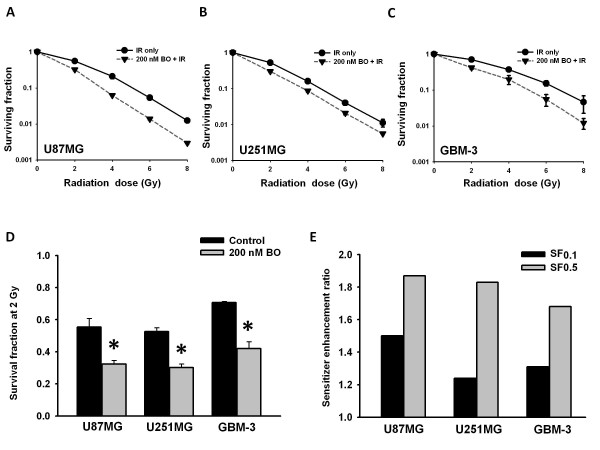
**The effect of BO-1051 on tumor cell radiosensitivity**. Cultures of (A) U87MG, (B) U251MG and (C) GBM-3 cells were exposed to 200 nM of BO-1051 or DMSO (IR only) for 24 h and irradiated with graded doses of γ-rays, rinsed, and fed with fresh growth media. Colony-forming efficiency was determined 10-14 days later, and survival curves were generated after normalizing for cell killing by BO-1051 alone. Points: mean survival fraction from at least 3 independent experiments; bars: SD. (D) The survival fraction after 2 Gy (SF_2_), corrected for independent cytotoxic effect of BO-1051, of human glioma cells treated with 200 nM of BO-1051 or control (DMSO) for 24 h pre-radiation was measured. Values are the mean survival fraction ± SD of at least 3 independent experiments. * p < 0.05. (E) Sensitizer enhancement ratios (SER) of human glioma cells. SERs were calculated at 10% or 50% cell survival (0.1 or 0.5) by dividing the dose of radiation from the radiation-only surviving curve with the corresponding dose from the BO-1051 plus radiation curve.

To investigate if BO-1051 enhances the cellular sensitivity to ionizing radiation, the glioma cells were exposed to BO-1051 for 24 h before irradiation and subjected to the clonogenic assay. The results showed that the SFs at different radiation dosages were apparently reduced in U87MG, U251MG and GBM-3 cells after they were exposed to BO-1051 (Figure. 2A-C). SFs after 2 Gy of BO-1051-pretreated cells were significantly lower than those of untreated cells (Figure 2D). Besides, the SERs were 1.50 for U87MG, 1.24 for U251MG and 1.31 for GBM-3 at a 10% cell survival (0.1). At 50% cell survival (0.5), the SERs were 1.87 for U87MG, 1.83 for U251MG and 1.68 for GBM-3 (Figure 2A-C, and 2E). As a result, the radiation survival curves obtained by the clonogenic assay showed that BO-1051 pretreatment sensitized human glioma cells to the ionizing radiation. Besides, Table 1 summarizes the relative reduction in SFs and compares them with a virtual value, expected for each of the combination of BO-1051 and irradiation dose. The actual SF measured for combinations is smaller than that expected on the basis of the treatment effects of each modality separately. It indicates a significant synergistic interaction in all three glioma cells.

**Table 1:** Relative reduction in surviving fraction of three glioma cells due to combination of irradiation and BO-1051 treatment

Irradiation dose (Gy)	Relative reduction (%)		
	
	U87MG	U251MG	GBM-3
2	41.7	42.6	40.6

4	70.4	45.6	47.0

6	74.2	47.9	64.3

8	76.3	50.0	73.9

Percentage relative reduction of the observed surviving fraction (SF) compared to the expected SF (calculated on the basis of combing individual treatment component, each with respective SF value).

### Induction of a G_2_/M phase arrest in glioma cells exposed to BO-1051

**Figure 3:**
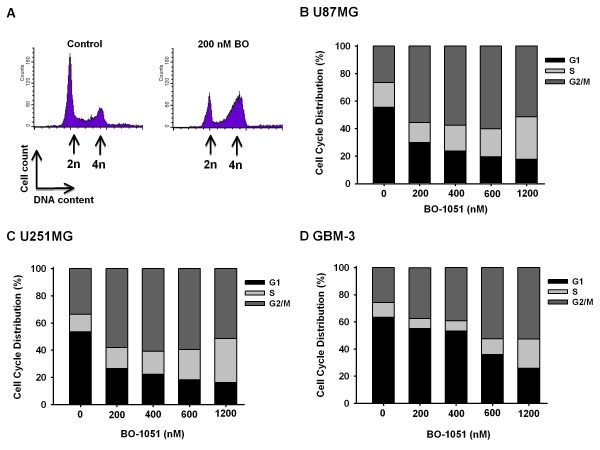
**Effect of BO-1051 on cell cycle profile in human glioma cells**. Cultures were exposed to BO-1051 for 24 h before collection and FACS analysis of the propidium iodide-stained cells. (A) The DNA histograms depict cell cycle phase distributions of U87MG 24 h post-treatment. Cells in exponential growth were sham treated (left panel), treated with BO-1051 (200 nM, right panel) and then harvested 24 h later. (B-D) Cell cycle distributions of a panel of 3 human glioma cell lines (U87MG, U251MG and GBM-3) were exposed to the designated concentrations of BO-1051 for 24 h. Data displayed by the DNA content profiles were analyzed, and the cell cycle phase information is represented graphically.

Given that radiosensitivity is distinct in different phases of the cell cycle, we tested the cell cycle distribution in BO-1051 treated glioma cells [22,23]. Cells were treated with BO-1051 for 24 h and then subjected to flow cytometric analysis. As illustrated in the DNA histograms, BO-1051 treatment significantly disturbed the cell cycle progression and showed a dramatic increase in G_2_/M phase populations in U87MG cells compared with the untreated controls (Figure 3A). Quantitative analysis of the cell-cycle distribution at 24 h post-exposure to BO-1051 at different concentrations from 200 nM to 1200 nM is shown in Figure 3B-D, which shows that G_2_/M phase arrest was caused by pre-treatment with BO-1051 in a dose-dependent manner for all 3 glioma cells (Figure 3A-D). Because the G_2_/M phase is known as the cell cycle's most radiosensitive phase [22,23], it may in part account for the effects of BO-1051 on the enhancement of radiosensitivity of glioma cell line.

### Enhancement of radiosensitivity by BO-1051 treatment is not caused by apoptosis or mitotic catastrophes in glioma cells

**Figure 4:**
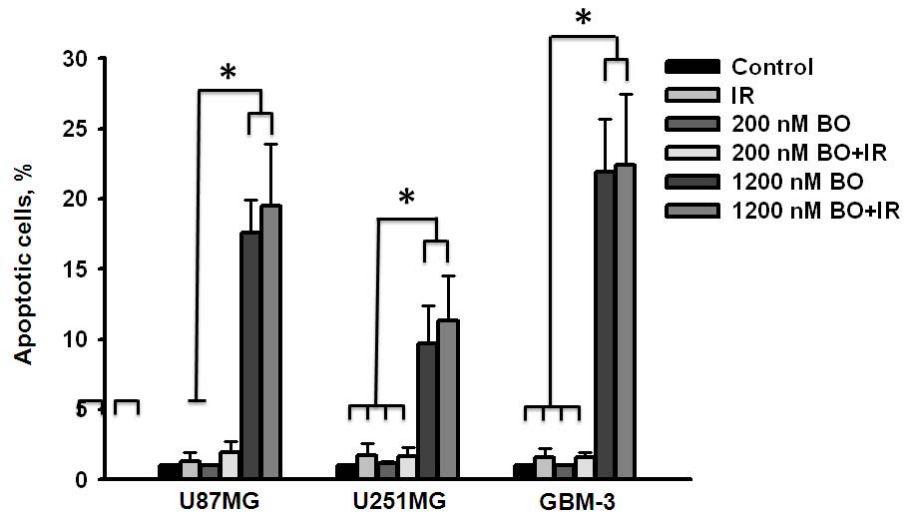
**Apoptotic effects of BO-1051 in combination with irradiation in glioma cells**. U87MG, U251MG and GBM-3 were exposed to 200 nM or higher concentration (1200 nM) of BO-1051 for 24 h and irradiated with 2 Gy, followed by FACS analysis of Annexin V-FITC and PI staining 24 h later. Control: no treatment; IR: ionizing radiation at 2 Gy; BO: BO-1051; BO+IR: cells exposed to BO-1051 for 24 h and then irradiation with 2 Gy of γ-ray. Values are the means ± SD of 3 independent experiments. * p < 0.05.

We next investigated whether BO-1051 enhanced radiation sensitivity of glioma cells was associated with increase of apoptosis. Cells were exposed to a range of BO-1051 concentrations (from 200 to 1200 nM) for 24 h, and then were irradiated with 2 Gy of γ-rays. The Annexin V/PI staining was then determined with FACS analysis. Cells treated with either 200 nM of BO-1051 alone or combined with irradiation exhibited less than 5% of apoptosis (Figure 4). Moreover, treatment with 1200 nM BO-1051 significantly induced approximately 20% of apoptosis in all 3 cell lines, but the combined protocol did not show obvious enhancement on the proportion of apoptotic cell deaths (Figure 4). An increase in radiosensitivity may be caused by radiation-induced mitotic catastrophes. Nevertheless, no significant mitotic catastrophes were detected in glioma cells treated with both BO-1051 and irradiation up to 72 h (unpublished data). These data indicate that the BO-1051-mediated increase in radiosensitivity is not due to the apoptosis and mitotic catastrophes.

### BO-1051 combined with γ-rays causes prolonged DNA damage response in glioma cells

**Figure 5:**
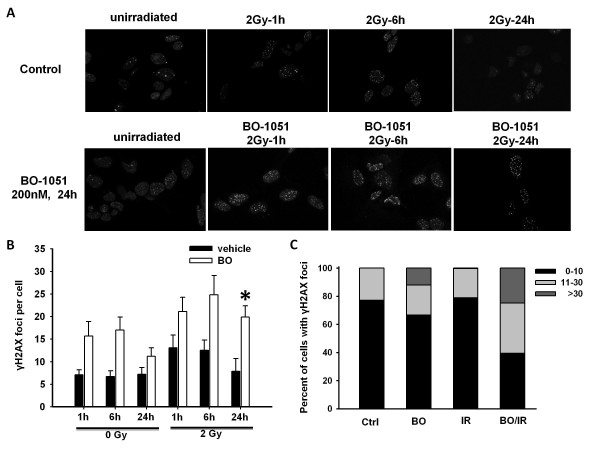
**Influence of BO-1051 on the repair of radiation-induced DSBs**. GBM-3 cells growing on slides in 35-mm dishes were exposed to 200 nM of BO-1051 for 24 h, irradiated (2Gy), and then fixed at the specified times for immunofluorescent analysis of nuclear γ-H2AX foci using a confocal microscope. (A) Immunofluorescent microscopy images of GBM-3 cells untreated or treated with 200 nM BO-1051 24 h before irradiation, fixed after 0, 1, 6, 24 h and then stained for γ-H2AX foci. (B) Quantitative analysis of γ-H2AX foci presented in irradiated cells following the above treatments. Filled columns: data from vehicle-treated cells; open columns: data from cells exposed to BO-1051. Values are the means ± SD of 3 independent experiments. * p < 0.05. (C) Distribution of γ-H2AX foci numbers per cell for one representative experiment at 24 h after irradiation. Ctrl: no treatment; IR: ironing radiation at 2 Gy; BO: cells exposed to 200 nM BO-1051; BO/IR: cells exposed to 200 nM BO-1051 for 24 h and then irradiated with 2 Gy of γ-rays. Foci were evaluated in 100 nuclei per treatment for each cell type. Values are the means at least of 3 independent experiments.

DNA damage is the most important biological effects caused by ionizing radiation. It has been reported that the nuclear foci of γ-H2AX is one of the canonical markers for evaluating the level of DNA damage [24]. To investigate if BO-1051 can affect the extent of DNA damage by γ-rays, the formation of γ-H2AX foci in cell nuclei was determined. Cells were treated with or without BO-1051 for 24 h prior to irradiation (2 Gy) and fed with BO-1051-free medium, and the average number of foci per cell was measured beginning at 1 h after irradiation and followed thereafter for 24 h. The results showed that exposure of glioma cells to either BO-1051 or irradiation (2 Gy) resulted in a significant increase of γ-H2AX foci at 1 h that was sustained for 6 h, and then the γ-H2AX foci declined to almost basal level at least 24 h after irradiation or drug removal (Figure 5A and 5B). The combined protocol resulted in a greater number of γ-H2AX foci than either of the individual treatments at 1 or 6 h. However, the number of residual γ-H2AX foci per cell 24 h post-irradiation was greater in BO-1051 plus irradiation (19.9 ± 2.5 per cell) compared with the number of foci in cells treated with either irradiation or BO-1051 alone (7.9 ± 2.8 and 11.2 ± 1.9 per cell, respectively) (Figure 5A and 5B). Furthermore, the frequency of γ-H2AX foci distribution at 24 h post-irradiation showed that the percentage of > 30 foci of γ-H2AX was higher than additive in BO-1051 plus irradiation (24.9%) compared with the percentage of foci in cells treated with either irradiation or BO-1051 alone (0.3% and 12.0%, respectively). These results suggest that BO-1051 produces supra-additive and prolonged effects of irradiation on glioma cells.

### BO-1051 delays the growth of xenograft gliomas exposed to irradiation

**Figure 6:**
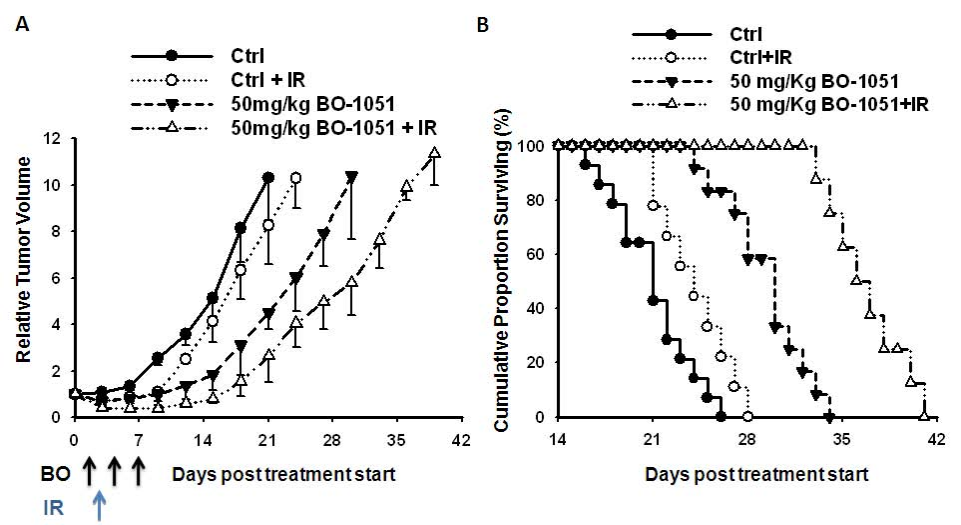
**The effects of BO-1051 on radiation-induced tumor growth delay and prolongation of TTF (time to treatment failure) in nude mice bearing GBM-3 xenografts**. When tumors reached 150 mm^3^, the nude mice with established GBM-3 flank xenografts were randomized into 4 groups: control (black circle), radiation (white circle), BO-1051 (black triangle) or BO-1051 plus radiation (white triangle). BO-1051 (50 mg/kg) was delivered via i.p. injection on days 0, 3 and 6, where day 0 begins on the day of randomization. Radiation (4 Gy) was delivered 24 h after the first injection of BO-1051 (day 1 after randomization), which corresponded to the same tumor size. Each group contained at least 8 mice. (A) Tumor growth rates for each treatment group were plotted as the mean relative tumor volume ± SD. Arrows indicate the time of BO-1051 and irradiation treatment. (B) Kaplan-Meier survival rates of nude mice with GBM-3 flank xenografts for each of the four treatments is depicted. Survival analysis was monitored daily. Treatment failure was defined as tumor size greater than 1500 mm^3 ^or the development of severe necrosis requiring euthanasia.

To determine if the enhanced radiosensitivity of BO-1051 treated glioma cells could be translated into an *in vivo *tumor model, a tumor growth delay assay using GBM-3 cells grown s.c. in the hind leg of nude mice was used. Mice bearing s.c. xenografts (~150 mm^3^) were stratified by size and randomized into 4 groups: control, BO-1051 alone, irradiation alone (4 Gy), or combined BO-1051 plus radiation. For the BO-1051 treatments, mice were i.p. injected with dosage at 50 mg/kg on days 0, 3 and 6. The growth rates for the GBM-3 tumors exposed to each treatment are shown in Figure 6A. For each group, the time for tumors to grow from 150 to 1500 mm^3 ^(i.e., a 10-fold increase in tumor size) was calculated using tumor volumes from the individual mice in each group (mean + SD). The time required for tumors to reach 10-times the starting volume increased from 20.2 days for control mice to 29.5 days for BO-1051-treated mice. Irradiation treatment alone increased the time to reach 10-times the initial volume to 23.6 days. However, in mice that received the combination therapy, the time for tumors to reach 10-times the initial volume increased to 36.2 days, which is significantly greater than the individual treatment groups (Figure 6A; Table 2, p > 0.05). Thus, the growth delay after the combined treatment was more than the sum of the growth delays caused by either BO-1051 or radiation alone. To calculate an SER comparing the tumor radiation responses in mice with and without the BO-1051 treatment, the normalized tumor growth delay was measured to determine the role of BO-1051 on tumor growth delay induced by the combination treatment. The SER of the xenograft gliomas was 1.97 with versus without the combined treatment of BO-1051 and irradiation (Table 2). Thus, BO-1051 alone slows tumor growth and enhances the effect of radiation, which is similar to the results obtained *in vitro*. Finally, the Kaplan-Meier survival curves of the combined treated mice revealed a trend toward longer survival in mice (Figure. 6B). We also noticed that the maximal toxicity of these agents decreased with body weight, and there was no more than a 15% weight reduction compared to the pretreatment body weight. However after cession of treatment, the body weight recovered (data not shown).

**Table 2:** BO-1051-induced tumor growth delay in GBM-3 xenografts

Treatment group	Tumor growth period, days*	Absolute growth delay†	Normalized growth delay‡	Enhancement ratio#
Control	20.2			

BO-1051	29.5	9.3		

IR	23.6	3.4		

BO-1051+IR	36.2	16	6.7	1.97

* Time for subcutaneous tumors to grow from the initial tumor volume to 10 times (see text).

† The number of days for the treated tumors to reach 10 times the initial tumor volume minus the number of days for the control group to reach the same size.

‡ The number of days for the tumors in the BO-1051+IR group to reach 10 times the initial tumor volume minus the number of days for tumors in the BO-1051-only group to reach the same size.

# Normalized growth delay for the BO-1051+IR group divided by absolute growth delay for the radiation-only group.

## Discussion

Although human GBM is one of the most radio-resistant tumors, radiotherapy remains routinely applied for patient treatment. Lots of efforts are made to develop methods for enhancing the radiosensitivity of GBM for promising therapy. Previous studies have shown that temozolomide (TMZ) combined with radiation exposure results in an increase of survival rate in a subset of human tumors [3,25,26]. Clinical studies also indicate that delivery of TMZ during radiotherapy increases survival rates of GBM patients, which suggests that this DNA alkylating agent can enhance the radiosensitivity of GBM [2,27,28]. Based on these previous studies, more efficient and safe DNA alkylating agents should be developed to increase the radiosensitivity in human GBM. Use of BO-1051 for cancer treatment has been supported by *in vitro *and *in vivo *preclinical studies [3,26]. The data presented here showed that the treatment of primary glioma cells and established cell lines with BO-1051 resulted in a dose-dependent induction of clonogenic cell death. It is supposed that BO-1051 can enhance the radiosensitivity via a synergistic effect since the survival fractions of combined treatment are lower than that of each individual treatment on glioma cell. However, additional studies are required to confirm that BO-1051 plays a synergistic or additive role on radiotherapy of gliomas. The anti-tumor and radiosensitizing effects of BO-1051 are encouraging because drugs showing efficacy against malignant glioma are still uncommon.

Bifunctional N-mustard alkylating agents, such as BO-1051, exhibits anticancer activity due to its ability to produce DNA interstrand and/or intrastrand cross-links [29,30]. As has been known, bifunctional alkylating agents induce collapsed replication forks that can lead to either cell cycle arrest, DNA repair, or apoptosis [31]. For example, the new synthesized alkylating agent BO-1012 shows anticancer activity on xenograft tumors that are formed by various human lung and bladder cancer cells [32]. BO-1051 and its analog(s) also exhibit similar behavior, and several related synthetic bifunctional N-mustards are under development [33]. Because BO-1051 contains the inherent lipophilicity for penetration through blood-brain barrier, it has efficiently demonstrated the ability to inhibit the growth of xenograft glioma in nude mice. Compared to other clinically used alkylating agents, such as melphalan and cisplatin, BO-1051 induced a higher level of ICLs [14]. BO-1051 also enhances the radiosensitivity of human glioma cell lines.

Although repair mechanisms such as homologous recombination and nonhomologous end-joining are important mammalian responses to double-strand DNA damage, cell cycle regulation is perhaps the most important determinant of irradiation sensitivity [22,34]. The cell cycle is strongly affected by DNA damage, and a cell's radiosensitivity depends on cell cycle position and progression [22]. Conventionally, the G_2_/M phase is the most radiosensitive phase compared to others. Several chemotherapeutic agents have been reported to enhance the radiosensitivity of cancer cells by accumulating the G_2_/M population, such as paclitaxel, indomethacin, 2-methoxyestradiol and TMZ [3,22,25,35-37]. From the results in this study, BO-1051 works by partially synchronized glioma cells in the most radiosensitive phase of the cell cycle, and it is suggested that BO-1051 may be a useful agent for adjuvant therapy on the glioma.

The phosphatidyl-inositol kinase-related protein ATM (ataxia-telangiectasia mutated), the most proximal signal transducer initiating cell cycle changes after the DNA damage/genomic stress [38], can be activated by BO-1051 in a dose-dependent manner in SAS cell line. It also activates the checkpoint kinase 2 (Chk2) in squamous cell carcinoma cell line after exposure to BO-1051 (unpublished observation). Chk2 activity is necessary for the phosphorylation of the dual-specificity phosphatases Cdc25A/C, which inactivates the enzymes, blocks CDK1 activation and causes a G_2 _arrest [39]. Furthermore, ATM's essential role in DNA damage and repair is highlighted by the extreme sensitivity to ionizing radiation of cells with defective ATM [40,41]. It, together with DNA-dependent protein kinase, phosphorylate the histone γ-H2AX foci, which can be visualized by immunofluorescence microscope as a discrete nuclear foci reflecting sites of DNA DSBs [42,43]. Although the specific relationship between the appearance of γ-H2AX foci and the repair of DSBs has not been completely defined, the reduction in the number of γ-H2AX foci in irradiated cells correlates with DNA repair, which is associated with the radiosensitivity [44-46]. It is also known that γ-H2AX is present in focal aggregates at sites of double-strand DNA damage and complex with other important repair molecules. γ-H2AX is required for foci formation for numerous factors including p53, MRN complex (MRE-11, RAD50, and NBS1), and BRCA1 [47]. MRN complex has also been implicated in the repair of small fraction of DSBs detectable as γ-H2AX foci that remain 24 h post-irradiation [48] Therefore, the observation that combined BO-1051 plus radiation significantly increased the levels of γ-H2AX foci. Because the prolonged expression of radiation-induced γ-H2AX foci may reflect the end result of disparate processes and events leading to maintenance of unrepaired DSBs, a distinctly different mechanism may be involved. Whereas the mechanism of this repair inhibition is not revealed in this investigation, additional investigations are required to define the molecular processes responsible for BO-1051-mediated radiosensitization.

Radiation sensitization could occur through any one of multiple modes of cell death. Zou et al. observed radiosensitization through the promotion of apoptosis [35], while another research group reported radiosensitization through a mitotic catastrophe [26,49] or senescence [50]. However, theses phenomenon were not detected in glioma cells exposed to BO-1051 following irradiation. Recently, we found that BO-1051 can induce autophagy in glioma cell lines (unpublished observation), and several lines of evidence have supported that autophagy is one of the causes of radiosensitization instead of apoptosis [51-53]. Therefore, the correlation between autophagy and radiosensitivity needs to be further investigated.

Given that human GBM usually exhibits high radioresistance, it is necessary to search for a specific radiosensitizer to enhance the radiosensitivity of GBM during radiotherapy. Kil et al. have demonstrated that TMZ may be used as a radiosensitizer because it can enhance the radiosensitivity of U251MG cells formed xenograft tumors [26]. Nevertheless, we found that TMZ was neither able to increase the radiosensitivity of xenograft tumors derived from GBM-3 cells nor able to delay tumor growth and improve animal survival after treatment (unpublished observation). Therefore, TMZ may exhibit cell specific effects for the treatment of different sources of human GBM. However, BO-1051 enhances the radiosensitivity of various glioma cell lines, as well as that of the corresponding xenograft tumors formed by GBM-3 cells. These results suggest that BO-1051 is a radiosensitizer with broader effects on different human GBM, and it may possess a clinical potential in the therapeutic strategy for treating malignant gliomas.

## Conclusions

GBM is the most malignant primary brain tumor in adults, but the effective therapeutic strategies remain under investigation. BO-1051 has been shown to inhibit the growth of gliomas. Here we further demonstrate that BO-1051 can significantly enhance the radiosensitivity. The enhanced radiosensitivity was found to be associated with G_2_/M phase arrest as well as the sustained DNA damage. *In vivo *studies further demonstrated that BO-1051 enhanced the radiotherapeutic effects on GBM-3-beared xenograft tumors. In this model, the combination of BO-1051 plus radiation produced the best response in terms of both local control and survival. These data suggest that BO-1051 provides a new strategy to improve therapeutic gain for radiation therapy.

## Abbreviations

BO-1051: 1-{4-[Bis(2-chloroethyl)amino]phenyl}-3-[2-methyl-5-(4-methylacridin-9-ylamino)phenyl]urea; DBSs: Double-strand breaks; GBM: Glioblastoma multiforme; ICLs: Interstrand cross-linking; PE: Plating efficacy; SER: Sensitizer enhancement ratio; SF: Surviving fraction; TMZ: Temozolomide; TTF: Time to treatment failure.

## Competing interests

The authors declare that they have no competing interest.

## Authors' contributions

PMC carried out most of the study, participated in its design, and drafted the manuscript. LHC, MTC, MHC, and YHS did parts of the statistical analysis and helped in discussion of data. YWC, SHY, PHT involved in drafting the manuscript in the section of radiotherapy techniques. HIM, SHC, TLS and YJL jointly conceived of the study, and coordination, participated in its design and drafted the manuscript. All authors read and approved the manuscript.

## References

[B1] Walker MD Green SB Byar DP Alexander E Jr Batzdorf U Brooks WH Hunt WE MacCarty CS Mahaley MS Jr Mealey J Jr Randomized comparisons of radiotherapy and nitrosoureas for the treatment of malignant glioma after surgery N Engl J Med 1980 303 23 1323 1329 10.1056/NEJM198012043032303 7001230

[B2] Stupp R Dietrich PY Ostermann Kraljevic S Pica A Maillard I Maeder P Meuli R Janzer R Pizzolato G Miralbell R Promising survival for patients with newly diagnosed glioblastoma multiforme treated with concomitant radiation plus temozolomide followed by adjuvant temozolomide J Clin Oncol 2002 20 5 1375 1382 10.1200/JCO.20.5.1375 11870182

[B3] Stupp R Mason WP van den Bent MJ Weller M Fisher B Taphoorn MJ Belanger K Brandes AA Marosi C Bogdahn U Radiotherapy plus concomitant and adjuvant temozolomide for glioblastoma N Engl J Med 2005 352 10 987 996 10.1056/NEJMoa043330 15758009

[B4] Wadhwa PD Zielske SP Roth JC Ballas CB Bowman JE Gerson SL Cancer gene therapy: scientific basis Annu Rev Med 2002 53 437 452 10.1146/annurev.med.53.082901.104039 11818484

[B5] Grando SA Kawashima K Wessler I Introduction: the non-neuronal cholinergic system in humans Life Sci 2003 72 18-19 2009 2012 10.1016/S0024-3205(03)00063-8 12628450

[B6] Maze R Carney JP Kelley MR Glassner BJ Williams DA Samson L Increasing DNA repair methyltransferase levels via bone marrow stem cell transduction rescues mice from the toxic effects of 1,3-bis(2-chloroethyl)-1-nitrosourea, a chemotherapeutic alkylating agent Proc Natl Acad Sci USA 1996 93 1 206 210 10.1073/pnas.93.1.206 8552605 PMC40207

[B7] Gravatt GL Baguley BC Wilson WR Denny WA DNA-directed alkylating agents. 6. Synthesis and antitumor activity of DNA minor groove-targeted aniline mustard analogues of pibenzimol (Hoechst 33258) J Med Chem 1994 37 25 4338 4345 10.1021/jm00051a010 7527862

[B8] Gourdie TA Valu KK Gravatt GL Boritzki TJ Baguley BC Wakelin LP Wilson WR Woodgate PD Denny WA DNA-directed alkylating agents. 1. Structure-activity relationships for acridine-linked aniline mustards: consequences of varying the reactivity of the mustard J Med Chem 1990 33 4 1177 1186 10.1021/jm00166a015 2319563

[B9] Bacherikov VA Chou TC Dong HJ Zhang X Chen CH Lin YW Tsai TJ Lee RZ Liu LF Su TL Potent antitumor 9-anilinoacridines bearing an alkylating N-mustard residue on the anilino ring: synthesis and biological activity Bioorg Med Chem 2005 13 12 3993 4006 10.1016/j.bmc.2005.03.057 15911312

[B10] Su TL Lin YW Chou TC Zhang X Bacherikov VA Chen CH Liu LF Tsai TJ Potent antitumor 9-anilinoacridines and acridines bearing an alkylating N-mustard residue on the acridine chromophore: synthesis and biological activity J Med Chem 2006 49 12 3710 3718 10.1021/jm060197r 16759114

[B11] Su TL Development of DNA topoisomerase II-mediated anticancer agents, 3-(9-acridinylamino)-5-hydroxymethylanilines (AHMAs) and related compounds Curr Med Chem 2002 9 18 1677 1688 12171550 10.2174/0929867023369231

[B12] Lee CH Chou TC Su TL Yu J Shao LE Yu AL BO-0742, a derivative of AHMA and N-mustard, has selective toxicity to drug sensitive and drug resistant leukemia cells and solid tumors Cancer Lett 2009 276 2 204 211 10.1016/j.canlet.2008.11.006 19108949

[B13] Kapuriya N Kapuriya K Dong H Zhang X Chou TC Chen YT Lee TC Lee WC Tsai TH Naliapara Y Novel DNA-directed alkylating agents: design, synthesis and potent antitumor effect of phenyl N-mustard-9-anilinoacridine conjugates via a carbamate or carbonate linker Bioorg Med Chem 2009 17 3 1264 1275 10.1016/j.bmc.2008.12.022 19124250

[B14] Kapuriya N Kapuriya K Zhang X Chou TC Kakadiya R Wu YT Tsai TH Chen YT Lee TC Shah A Synthesis and biological activity of stable and potent antitumor agents, aniline nitrogen mustards linked to 9-anilinoacridines via a urea linkage Bioorg Med Chem 2008 16 10 5413 5423 10.1016/j.bmc.2008.04.024 18450456

[B15] Kim JH Shin JH Kim IH Susceptibility and radiosensitization of human glioblastoma cells to trichostatin A, a histone deacetylase inhibitor Int J Radiat Oncol Biol Phys 2004 59 4 1174 1180 10.1016/j.ijrobp.2004.03.001 15234053

[B16] Camphausen K Brady KJ Burgan WE Cerra MA Russell JS Bull EE Tofilon PJ Flavopiridol enhances human tumor cell radiosensitivity and prolongs expression of gammaH2AX foci Mol Cancer Ther 2004 3 4 409 416 15078984

[B17] McCord AM Jamal M Williams ES Camphausen K Tofilon PJ CD133+ glioblastoma stem-like cells are radiosensitive with a defective DNA damage response compared with established cell lines Clin Cancer Res 2009 15 16 5145 5153 10.1158/1078-0432.CCR-09-0263 19671863 PMC6290462

[B18] Dote H Cerna D Burgan WE Carter DJ Cerra MA Hollingshead MG Camphausen K Tofilon PJ Enhancement of *in vitro *and *in vivo *tumor cell radiosensitivity by the DNA methylation inhibitor zebularine Clin Cancer Res 2005 11 12 4571 4579 10.1158/1078-0432.CCR-05-0050 15958643

[B19] Barker CA Burgan WE Carter DJ Cerna D Gius D Hollingshead MG Camphausen K Tofilon PJ *In vitro *and *in vivo *radiosensitization induced by the ribonucleotide reductase inhibitor Triapine (3-aminopyridine-2-carboxaldehyde-thiosemicarbazone) Clin Cancer Res 2006 12 9 2912 2918 10.1158/1078-0432.CCR-05-2860 16675588

[B20] Russell JS Burgan W Oswald KA Camphausen K Tofilon PJ Enhanced cell killing induced by the combination of radiation and the heat shock protein 90 inhibitor 17-allylamino-17- demethoxygeldanamycin: a multitarget approach to radiosensitization Clin Cancer Res 2003 9 10 Pt 1 3749 3755 14506167

[B21] Geldof AA Slotman BJ Radiosensitizing effect of cisplatin in prostate cancer cell lines Cancer Lett 1996 101 2 233 239 10.1016/0304-3835(96)04140-7 8620475

[B22] Pawlik TM Keyomarsi K Role of cell cycle in mediating sensitivity to radiotherapy Int J Radiat Oncol Biol Phys 2004 59 4 928 942 10.1016/j.ijrobp.2004.03.005 15234026

[B23] Sinclair WK Morton RA X-ray sensitivity during the cell generation cycle of cultured Chinese hamster cells Radiat Res 1966 29 3 450 474 10.2307/3572025 5924188

[B24] Pilch DR Sedelnikova OA Redon C Celeste A Nussenzweig A Bonner WM Characteristics of gamma-H2AX foci at DNA double-strand breaks sites Biochem Cell Biol 2003 81 3 123 129 10.1139/o03-042 12897845

[B25] Chakravarti A Erkkinen MG Nestler U Stupp R Mehta M Aldape K Gilbert MR Black PM Loeffler JS Temozolomide-mediated radiation enhancement in glioblastoma: a report on underlying mechanisms Clin Cancer Res 2006 12 15 4738 4746 10.1158/1078-0432.CCR-06-0596 16899625

[B26] Kil WJ Cerna D Burgan WE Beam K Carter D Steeg PS Tofilon PJ Camphausen K *In vitro *and *in vivo *radiosensitization induced by the DNA methylating agent temozolomide Clin Cancer Res 2008 14 3 931 938 10.1158/1078-0432.CCR-07-1856 18245557

[B27] Kanzawa T Germano IM Komata T Ito H Kondo Y Kondo S Role of autophagy in temozolomide-induced cytotoxicity for malignant glioma cells Cell Death Differ 2004 11 4 448 457 10.1038/sj.cdd.4401359 14713959

[B28] Yung WK Albright RE Olson J Fredericks R Fink K Prados MD Brada M Spence A Hohl RJ Shapiro W A phase II study of temozolomide vs. procarbazine in patients with glioblastoma multiforme at first relapse Br J Cancer 2000 83 5 588 593 10.1054/bjoc.2000.1316 10944597 PMC2363506

[B29] Tomasz M Palom Y The mitomycin bioreductive antitumor agents: cross-linking and alkylation of DNA as the molecular basis of their activity Pharmacol Ther 1997 76 1-3 73 87 10.1016/S0163-7258(97)00088-0 9535170

[B30] Panasci L Xu ZY Bello V Aloyz R The role of DNA repair in nitrogen mustard drug resistance Anticancer Drugs 2002 13 3 211 220 10.1097/00001813-200203000-00002 11984064

[B31] Sorensen CS Hansen LT Dziegielewski J Syljuasen RG Lundin C Bartek J Helleday T The cell-cycle checkpoint kinase Chk1 is required for mammalian homologous recombination repair Nat Cell Biol 2005 7 2 195 201 10.1038/ncb1212 15665856

[B32] Kakadiya R Dong H Lee PC Kapuriya N Zhang X Chou TC Lee TC Kapuriya K Shah A Su TL Potent antitumor bifunctional DNA alkylating agents, synthesis and biological activities of 3a-aza-cyclopenta[a]indenes Bioorg Med Chem 2009 17 15 5614 5626 10.1016/j.bmc.2009.06.018 19576785

[B33] Kakadiya R Dong H Kumar A Narsinh D Zhang X Chou TC Lee TC Shah A Su TL Potent DNA-directed alkylating agents: Synthesis and biological activity of phenyl N-mustard-quinoline conjugates having a urea or hydrazinecarboxamide linker Bioorg Med Chem 2010 18 6 2285 2299 10.1016/j.bmc.2010.01.061 20181487

[B34] Shrivastav M De Haro LP Nickoloff JA Regulation of DNA double-strand break repair pathway choice Cell Res 2008 18 1 134 147 10.1038/cr.2007.111 18157161

[B35] Zou H Zhao S Zhang J Lv G Zhang X Yu H Wang H Wang L Enhanced radiation-induced cytotoxic effect by 2-ME in glioma cells is mediated by induction of cell cycle arrest and DNA damage via activation of ATM pathways Brain Res 2007 1185 231 238 10.1016/j.brainres.2007.07.092 17980860

[B36] Jin C Wu H Liu J Bai L Guo G The effect of paclitaxel-loaded nanoparticles with radiation on hypoxic MCF-7 cells J Clin Pharm Ther 2007 32 1 41 47 10.1111/j.1365-2710.2007.00796.x 17286788

[B37] Furuta Y Hunter N Barkley T Jr Hall E Milas L Increase in radioresponse of murine tumors by treatment with indomethacin Cancer Res 1988 48 11 3008 3013 3365690

[B38] Samuel T Weber HO Funk JO Linking DNA damage to cell cycle checkpoints Cell Cycle 2002 1 3 162 168 10.4161/cc.1.3.118 12429926

[B39] Herzinger T Funk JO Hillmer K Eick D Wolf DA Kind P Ultraviolet B irradiation-induced G2 cell cycle arrest in human keratinocytes by inhibitory phosphorylation of the cdc2 cell cycle kinase Oncogene 1995 11 10 2151 2156 7478536

[B40] Kastan MB Lim DS The many substrates and functions of ATM Nat Rev Mol Cell Biol 2000 1 3 179 186 10.1038/35043058 11252893

[B41] Collis SJ Swartz MJ Nelson WG DeWeese TL Enhanced radiation and chemotherapy-mediated cell killing of human cancer cells by small inhibitory RNA silencing of DNA repair factors Cancer Res 2003 63 7 1550 1554 12670903

[B42] Rogakou EP Pilch DR Orr AH Ivanova VS Bonner WM DNA double-stranded breaks induce histone H2AX phosphorylation on serine 139 J Biol Chem 1998 273 10 5858 5868 10.1074/jbc.273.10.5858 9488723

[B43] Sedelnikova OA Rogakou EP Panyutin IG Bonner WM Quantitative detection of (125)IdU-induced DNA double-strand breaks with gamma-H2AX antibody Radiat Res 2002 158 4 486 492 10.1667/0033-7587(2002)158[0486:QDOIID]2.0.CO;2 12236816

[B44] Olive PL Banath JP Phosphorylation of histone H2AX as a measure of radiosensitivity Int J Radiat Oncol Biol Phys 2004 58 2 331 335 10.1016/j.ijrobp.2003.09.028 14751500

[B45] Nazarov IB Smirnova AN Krutilina RI Svetlova MP Solovjeva LV Nikiforov AA Oei SL Zalenskaya IA Yau PM Bradbury EM Dephosphorylation of histone gamma-H2AX during repair of DNA double-strand breaks in mammalian cells and its inhibition by calyculin A Radiat Res 2003 160 3 309 317 10.1667/RR3043 12926989

[B46] Banath JP Macphail SH Olive PL Radiation sensitivity, H2AX phosphorylation, and kinetics of repair of DNA strand breaks in irradiated cervical cancer cell lines Cancer Res 2004 64 19 7144 7149 10.1158/0008-5472.CAN-04-1433 15466212

[B47] Bilsland E Downs JA Tails of histones in DNA double-strand break repair Mutagenesis 2005 20 3 153 163 10.1093/mutage/gei031 15843385

[B48] Lobrich M Jeggo PA Harmonising the response to DSBs: a new string in the ATM bow DNA Repair (Amst) 2005 4 7 749 759 10.1016/j.dnarep.2004.12.008 15978533

[B49] Mitchell JB Choudhuri R Fabre K Sowers AL Citrin D Zabludoff SD Cook JA *In vitro *and *in vivo *radiation sensitization of human tumor cells by a novel checkpoint kinase inhibitor, AZD7762 Clin Cancer Res 2010 16 7 2076 2084 10.1158/1078-0432.CCR-09-3277 20233881 PMC2851146

[B50] Lehmann BD McCubrey JA Terrian DM Radiosensitization of Prostate Cancer by Priming the Wild-Type p53-Dependent Cellular Senescence Pathway Cancer Biol Ther 2007 6 8 1165 1170 18059157 10.4161/cbt.6.8.4544 PMC2889025

[B51] Fujiwara K Iwado E Mills GB Sawaya R Kondo S Kondo Y Akt inhibitor shows anticancer and radiosensitizing effects in malignant glioma cells by inducing autophagy Int J Oncol 2007 31 4 753 760 17786305

[B52] Kim KW Hwang M Moretti L Jaboin JJ Cha YI Lu B Autophagy upregulation by inhibitors of caspase-3 and mTOR enhances radiotherapy in a mouse model of lung cancer Autophagy 2008 4 5 659 668 18424912 10.4161/auto.6058 PMC3073356

[B53] Tsuboi Y Kurimoto M Nagai S Hayakawa Y Kamiyama H Hayashi N Kitajima I Endo S Induction of autophagic cell death and radiosensitization by the pharmacological inhibition of nuclear factor-kappa B activation in human glioma cell lines J Neurosurg 2009 110 3 594 604 10.3171/2008.8.JNS17648 19046042

